# Intraprosthetic dislocation of dual-mobility total hip arthroplasty implant

**DOI:** 10.1016/j.radcr.2023.08.070

**Published:** 2023-08-30

**Authors:** Perry Veras, Patrick Gidley, Nisha R. Patel, Caleb Bhatnagar, Rami El-Baba, Emad Allam

**Affiliations:** Loyola University Medical Center and Loyola University Chicago, 2160 S First Ave, Maywood, IL, 60153, USA

**Keywords:** Total hip arthroplasty, Dual mobility, Hardware complications, Intraprosthetic dislocation, Polyethylene

## Abstract

Dual-mobility total hip arthroplasties were developed to decrease the risk of dislocation and instability seen with traditional fixed-bearing total hip arthroplasties. However, dual-mobility constructs, notably the first-generation design, come with a risk of intraprosthetic dislocation (IPD). These dislocations occur when the polyethylene femoral head component is dislodged, causing direct articulation between the inner ceramic femoral head and the metal acetabular shell. This is different than a polyethylene liner dislocation in a standard total hip arthroplasty. Causes of IPD include polyethylene wear and iatrogenic dislocation from closed reduction attempts. Timely identification is essential to reduce the risk of soft tissue metallosis, raised cobalt and chromium levels, and the need for major revisions. This complication can be seen on imaging, but radiologists must be aware of the various components and mechanisms of failure to recognize this unique complication. We present a case of a dual-mobility construct with IPD between the femoral head components, illustrated on radiographs and CT and subsequently confirmed at the time of surgery.

## Introduction

**Fig. 1:**
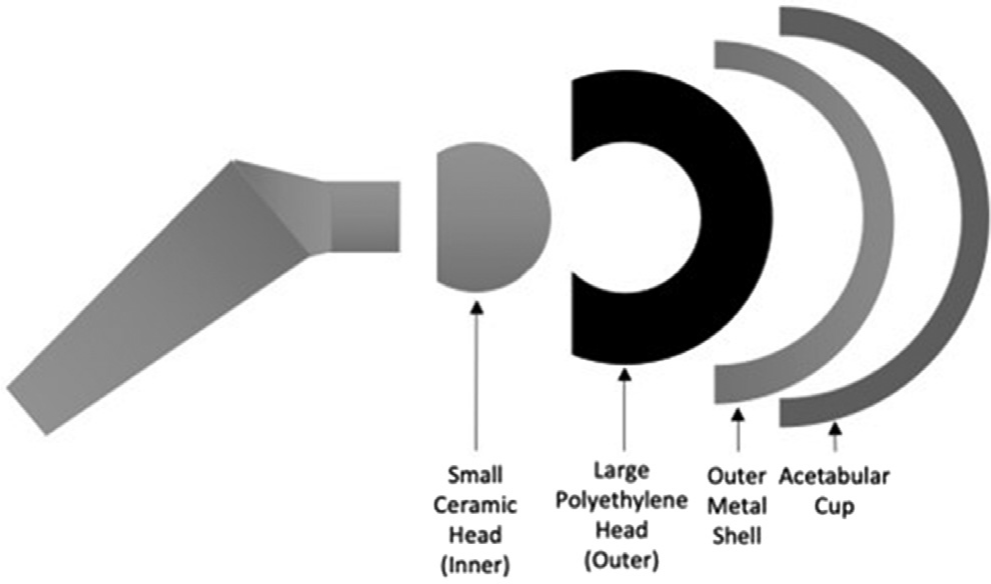
Component configuration of dual-mobility total hip arthroplasty.

More than 450,000 total hip arthroplasty (THA) procedures are performed in the United States each year, with numbers continuing to grow [1]. Instability and dislocation are among the most common complications of THA [1,2]. Developments to minimize dislocation risk include larger femoral head diameters, constrained acetabular components, and dual-mobility implants. Research has shown that dual-mobility implants reduce the dislocation rate after primary and revision THA [3]. These implants are constructed with a small inner ceramic femoral head component that articulates but is constrained within the larger outer polyethylene femoral head. The polyethylene head, sometimes called the polyethylene liner, then articulates unconstrained within the outer metal acetabular shell (Fig. 1) [4].

While dual-mobility implants carry a lower risk, dislocation is still possible. The construction of the dual-mobility implant lends it to the possible dislocation of the polyethylene component from its housing in the metal shell and from the ceramic femoral head, also known as intraprosthetic dislocation [4]. Recent studies have shown an incidence of IPD ranging from 0% to 0.3% [5,6].

Though IPD of dual-mobility implants is uncommon, this type of hardware failure is vital to identify on imaging as they can cause further issues. We have identified only one other case in the literature illustrating IPD on radiographs and CT [9].

## Case presentation

The patient was a 69-year-old male with a history of hypertension, hyperlipidemia, diabetes mellitus, and a left THA complicated by multiple dislocations. He presented to the orthopedic surgery clinic for left hip evaluation. He also reported a small palpable mass in his left posterior hip. He had left lower extremity foot drop and pain at baseline due to neuropathy but did not report numbness or paresthesia. One year prior, the patient underwent conversion of his left THA to a dual mobility hip arthroplasty due to dislocation of his left THA and frequent instability. Since the conversion, he had experienced 2 subsequent hip dislocation events.

**Fig. 2:**
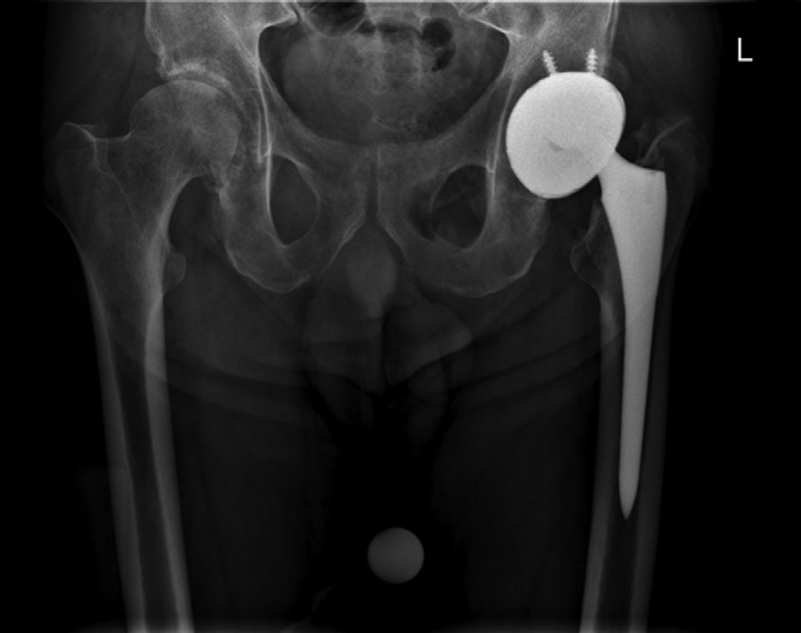
Low-centered AP radiograph of the pelvis shows a left total hip arthroplasty. There is eccentric positioning of the femoral head component within the superior aspect of the acetabular cup.

**Fig. 3:**
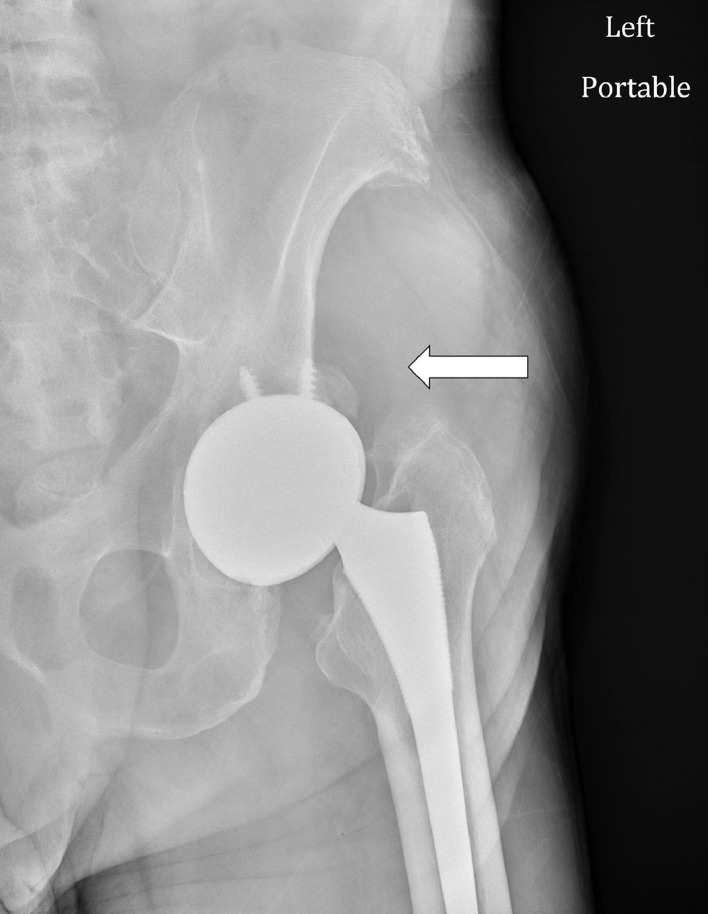
AP radiograph of the left hip shows a subtle semicircular lucency along the superolateral aspect of the hip (arrow) indicative of the “bubble sign.” There is a total hip arthroplasty without acute fracture, hip dislocation, periprosthetic lucency, or soft tissue gas. There is minimal heterotopic ossification along the superolateral left acetabulum.

Upon presentation to the orthopedic clinic, history and radiographs revealed concerns for IPD. On radiographs, there was eccentric positioning of the femoral head component in the acetabular cup, and a subtle adjacent semicircular lucency suggestive of displaced polyethylene (Figs. 2 and 3). Therefore, a CT of the left hip was obtained.

**Fig. 4:**
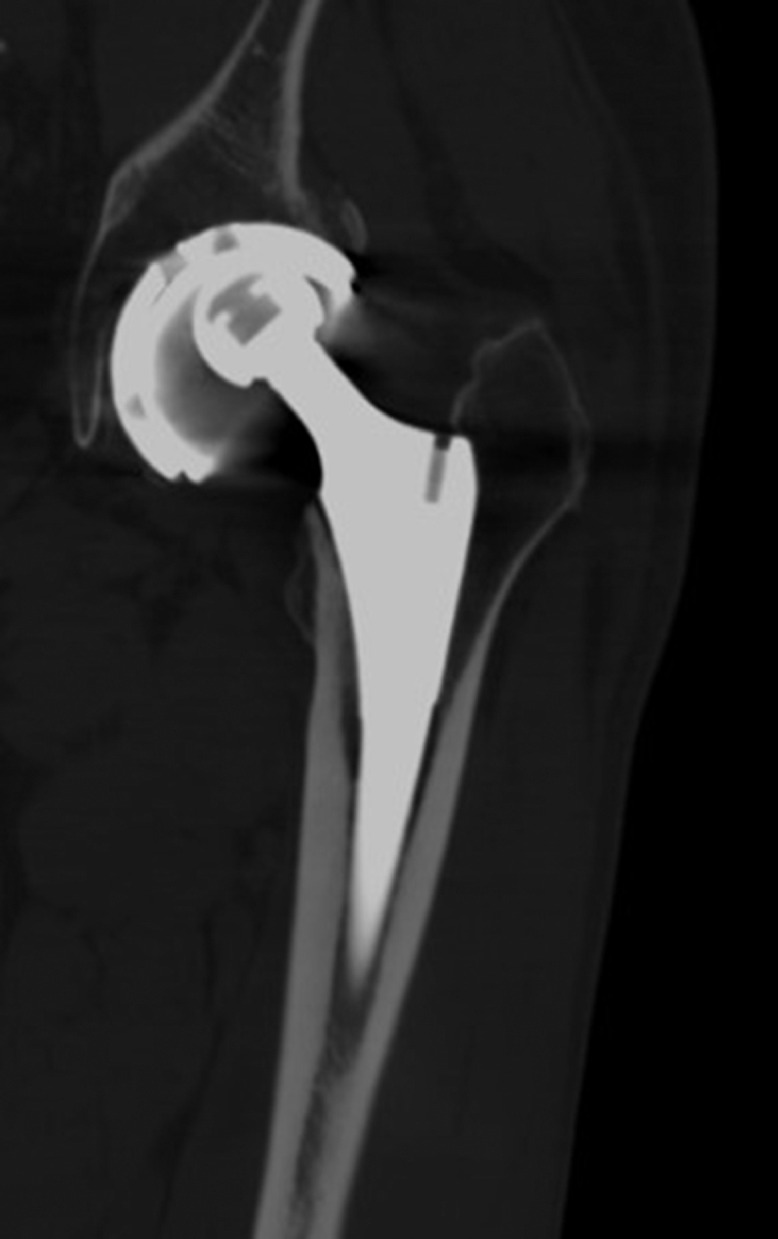
Coronal CT image of the left hip confirms eccentric positioning of the ceramic femoral head within the superior aspect of the metal shell and acetabular cup assembly. In this CT image, the ceramic femoral head articulates with the metal shell without an intervening polyethylene femoral head component. The metal shell is within the acetabular cup.

**Fig. 5:**
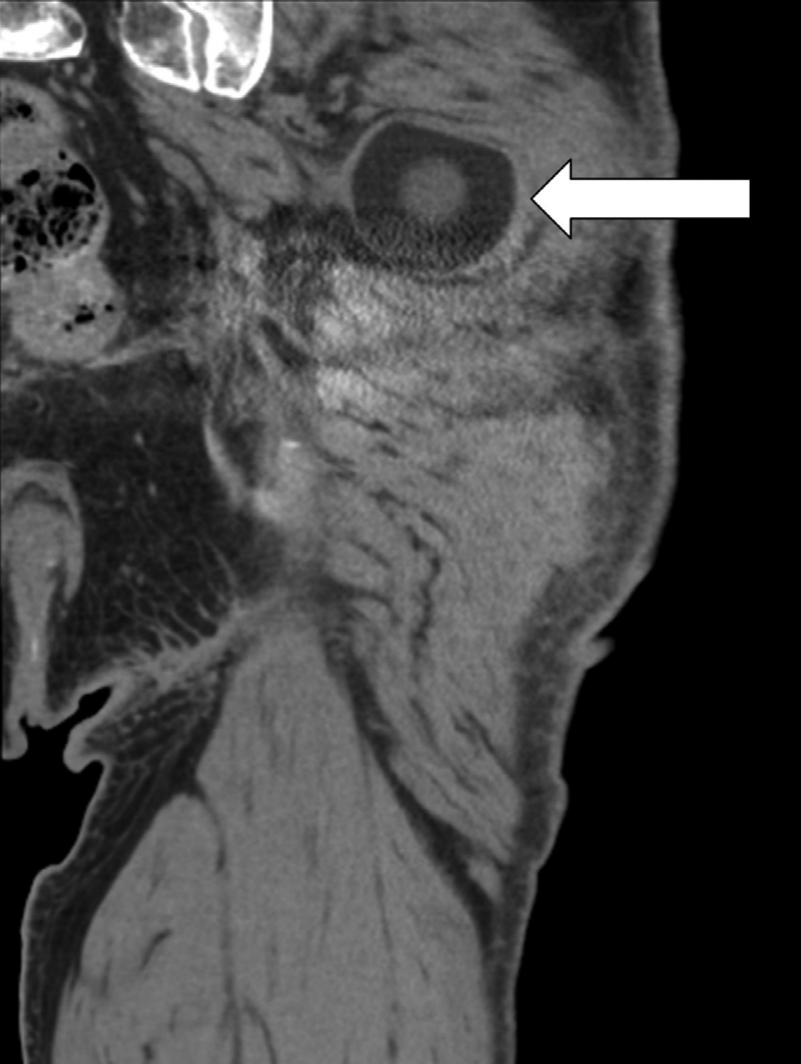
Coronal CT image of the left hip (posterior relative to the previous coronal CT image) shows that the polyethylene head (arrow) is displaced in the posterior gluteal musculature.

**Fig. 6:**
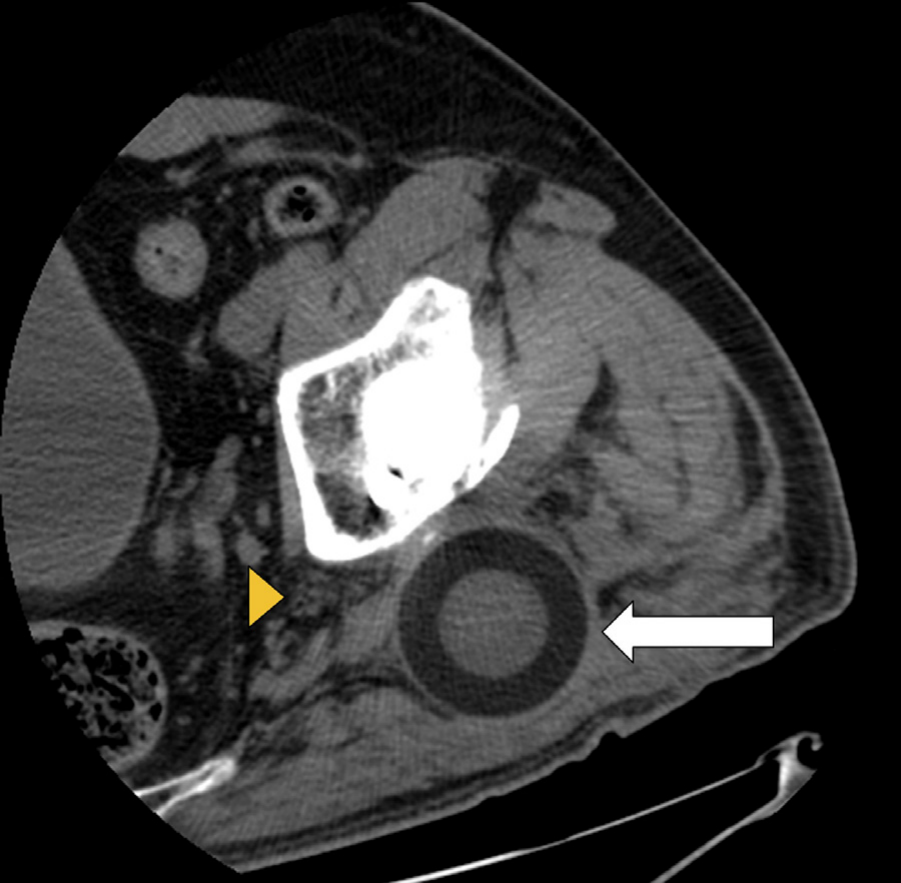
Axial CT image also shows that the polyethylene head (arrow) is displaced in the posterior gluteal musculature. The displaced head does not definitively contact the sciatic nerve (arrowhead) on this CT obtained with the patient lying supine.

**Fig. 7:**
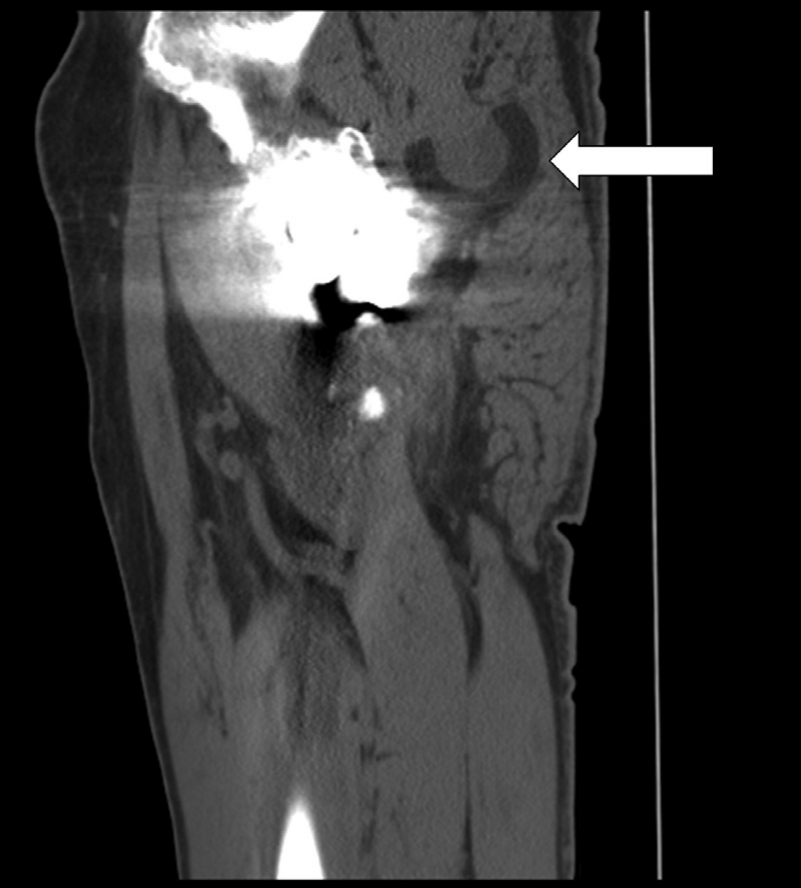
Sagittal CT image confirms that the polyethylene femoral head (arrow) is dislocated posterosuperior in the posterior gluteal musculature. The polyethylene head is inverted. The polyethylene appears intact without focal thinning or fragmentation.

The CT demonstrated a ceramic femoral head component articulating directly with the dual mobility metal liner; the intervening polyethylene femoral head was separated and displaced in the posterior gluteal musculature (Fig. 4, Fig. 5, Fig. 6, Fig. 7). These findings were consistent with IPD. This was confirmed during subsequent surgery. The polyethylene was retrieved. The hip arthroplasty was revised from a dual mobility construct to a constrained construct.

## Discussion

Intraprosthetic hip dislocation often presents with limping, leg shortening, and grinding sensations in the affected limb [7]. Our patient presented with a small palpable mass in his left posterior hip. Plain radiography can raise suspicion for IPD by showing eccentric positioning of the prosthesis or displacement of the polyethylene head outside the acetabular cup, also known as a “bubble sign” (Fig. 3) [8]. Abnormal radiographs or clinical concerns should prompt advanced imaging. Adjusting windows/levels on CT is critical for visualizing the different components and limiting metal artifacts. CT imaging can demonstrate the position of the dislocated polyethylene component and provide information for surgical planning [9]. The polyethylene head can migrate into the soft tissues, as seen in our patient, or deeper into the pelvis [10].

Polyethylene liner wear is the most common cause of IPD [4]. Another common cause of IPD is iatrogenic dislocation due to closed reduction of a polyethylene head-metal shell dislocation. This is likely the mechanism causing IPD in this patient. During closed reduction, the polyethylene head might be caught at the edge of the metal shell or bony pelvic prominence, causing dissociation of the ceramic head-polyethylene head capture mechanism, known as the bottle-opener effect [8]. Thus, it is critical to identify if a patient has a dual-mobility implant prior to closed reduction attempts to avoid the bottle-opener effect. Proper sedation and muscle relaxation or general anesthesia are needed when resetting polyethylene head-metal shell dislocations [11]. Correlation with surgical timeline or prior imaging is helpful to differentiate the cause of dislocation, as liner wear typically develops over the years, whereas liner dislocation can occur acutely [12].

Displacement of the polyethylene head can cause direct articulation between the ceramic head and the metal shell. Prolonged articulation between these components can cause damage to the components and may result in significant soft tissue metallosis and raised cobalt and chromium levels [7,13]. Thus, early detection of IPD is paramount to avoiding severe consequences or the need for major revision procedures.

## Conclusion

We presented a patient with a history of dual-mobility THA who complained of a palpable mass in his left posterior hip. Imaging illustrated the IPD of the polyethylene liner, and surgery successfully retrieved the dislocated polyethylene component and revised the THA. Although rare, the unique configuration of dual-mobility THA lends itself to the unique complication of IPD. Prolonged direct articulation between the ceramic head and the metal shell can lead to complications such as soft tissue metallosis, raised cobalt and chromium levels, and the need for major revisions. Therefore, radiologists and surgeons must be familiar with dual-mobility THA components and recognize this mode of hardware failure on imaging when cases arise.

## Patient consent

Informed consent for this case was obtained from the patient.
